# Frailty efficacy as a predictor of clinical and cognitive complications in patients undergoing coronary artery bypass grafting: a prospective cohort study

**DOI:** 10.1186/s12872-024-03781-7

**Published:** 2024-02-16

**Authors:** Mehrnoosh Bakhtiari, Farhad Shaker, Fatemeh Ojaghi Shirmard, Arash Jalali, Ahmad Vakili-Basir, Mohammad Balabandian, Sima Shamshiri Khamene, Izat Mohammadkhawajah, Akbar Shafiee, Seyedeh Zahra Badrkhahan, Kaveh Hosseini

**Affiliations:** 1 Cardiac Primary Prevention Research Center, Cardiovascular Diseases Research Institute, Tehran University of Medical Sciences, Tehran, Iran; 2 Tehran Heart Center, Cardiovascular Diseases Research Institute, Tehran University of Medical Sciences, North Kargar Ave, Tehran, Iran; 3 Department of Epidemiology and Biostatistics, School of Public Health, Tehran University of Medical Sciences, Tehran, Iran; 4 School of Medicine, Iran University of Medical Sciences, Tehran, Iran; 5 Department of Geriatric Medicine, Tehran University of Medical Sciences (TUMS), Tehran, Iran

**Keywords:** Frailty, Coronary artery bypass grafting, Cognitive function, Independence level, Depression status

## Abstract

**Background:**

Frailty is proposed as a predictor of outcomes in patients undergoing major surgeries, although data on the association of frailty and coronary artery bypass grafting (CABG) are lacking. We assessed the association between frailty and cognitive and clinical complications following CABG.

**Methods:**

This prospective study included patients aged over 60 years undergoing elective CABG at Tehran Heart Center from 2020 to 2022. Baseline and three-month follow-up data on frailty using the Frail scale and clinical Frail scale, functional status using the Lawton Instrumental Activities of Daily Living Scale (IADL), cognitive function by Montreal Cognitive Assessment (MoCA), and depression by the Geriatric Depression Scale (GDS) were obtained. The incidence of adverse outcomes was investigated at the three-month follow-up. Outcomes between frail and non-frail groups were compared utilizing T-tests and Mann-Whitney U tests, as appropriate.

**Results:**

We included 170 patients with a median age of 66 ± 4 years (75.3% male). Of these, 58 cases were classified as frail, and 112 individuals were non-frail, preoperatively. Frail patients demonstrated significantly worse baseline MOCA scores (21.08 vs. 22.41, *P* = 0.045), GDS (2.00 vs. 1.00, *P* = 0.009), and Lawton IADL (8.00 vs. 6.00, *P* < 0.001) compared to non-frail. According to 3-month follow-up data, postoperative MOCA and GDS scores were comparable between the two groups, while Lawton IADL (8.00 vs. 6.00, *P* < 0.001) was significantly lower in frail cases. A significantly higher rate of readmission (1.8% vs. 12.1%), sepsis (7.1% vs. 19.0%), as well as a higher Euroscore (1.5 vs. 1.9), was observed in the frail group. A mildly significantly more extended ICU stay (6.00 vs. 5.00, *p* = 0.051) was shown in the frail patient.

**Conclusion:**

Frailty showed a significant association with a worse preoperative independence level, cognitive function, and depression status, as well as increased postoperative complications.

**Supplementary Information:**

The online version contains supplementary material available at 10.1186/s12872-024-03781-7.

## Introduction

Coronary artery bypass graft (CABG) is one of the most common cardiac surgeries in patients with severe coronary disease and myocardial ischemia, with an incidence rate of 62 per 100,000 residents in Europe and nearly 200,000 cases annually in the USA [1]. However, while a substantial decrease trend in post-discharge and in-hospital mortality has been shown in patients undergoing CABG, a considerable major complications incidence rate of 41% has been reported in these cases [2, 3], which accentuates the importance of identifying prognostic factors to identify high-risk patients. Although age is an important prognostic factor and the worst clinical outcomes, such as higher mortality and pulmonary and cardiac complications, have been shown in elderly patients [4], selecting patients before surgery based on age may no longer be helpful as many non-elderly patients show frailty features and have less desirable hospital yields [5].

Frailty, with a prevalence ranging from 10 to 60% in cardiovascular patients, could be defined as a decrease in resilience to stressors due to insufficient physiological supply, mainly in elderly patients [6, 7]. Several mechanisms contribute to frailty development, such as poor nutritional support, physical dormancy, and cognitive decline, as well as comorbidities such as hypertension and diabetes mellitus [6, 8]. Additionally, in recent studies, Frailty has been shown to be associated with increasing mortality, length of stay in the hospital, longer intensive care unit (ICU) stay, and more discharges to intermediate care facilities in patients undergoing cardiac surgeries [9]. A recent study demonstrated that frailty was associated with an increased risk of cognitive impairment, depression, and chronic depression in patients with cardiovascular diseases [10]. Moreover, the role of frailty in predicting worse functional and cognitive outcomes in cardiac surgery has been previously highlighted [11, 12].

The lack of sufficient evidence related to cardiac surgery outcomes and associated factors in the elderly population of Iran has been previously mentioned [13], highlighting the necessity of conducting relevant studies. To the best of our knowledge, the association of frailty with cognitive dysfunction exclusively after CABG has not been widely investigated, and we aimed to investigate frailty as a predictive factor, assisting clinicians in risk stratification and preoperative counseling. This study aims to investigate the prognostic role of frailty in clinical complications, independence level, and cognitive impairment following CABG in frail patients compared to non-frail subjects.

## Methods

Study procedures were approved by the Tehran Heart Center research ethics committee (IR.TUMS.THC.REC.1400.005). Written informed consent was obtained from patients. For illiterate patients, informed consent was obtained from the patient’s legal guardian. All cognitive and physical data were gathered using a predefined questionnaire, and no interventions were administered during the study. Patient health and personal information were treated with confidentiality. Patients had the option to withdraw from the study at any point without affecting their medical care.

### Study design

We conducted a prospective cohort study involving 170 patients aged 60 and older who underwent elective coronary artery bypass grafting (CABG) at Tehran Heart Center between 2020 and 2022. Relevant data, including echocardiography, angiography, and laboratory results, were collected before surgery. Perioperative assessments of frailty, cognitive and executive function, and depression were performed by a cardiology resident or geriatrics physician. Postoperative outcomes, such as atrial fibrillation (AF), sepsis, pneumonia, and hospitalization duration, were evaluated. Follow-up assessments were conducted after three months to reevaluate frailty, functional performance, cognitive status, and additional postoperative outcomes. The sample size calculation was carried out using G Power based on the prevalence of frailty observed in the study by Nakano et al. The effect size was pre-set at 0.53, aiming for a statistical power of 0.9 and maintaining a significance level (α) of 0.05, and according to these parameters, our cohort required 170 patients, about 57 frail and 113 non-frail subjects.

### Inclusion and exclusion criteria

We included all patients with the following criteria: (1) patients aged 60 years or more. (2) patients with elective CABG surgery, (3) patients with no other simultaneous surgery during CABG.

patients with the following criteria were excluded from the study: (1) patients aged less than 60 years, (2) patients for cardiac surgeries other than CABG, and (3) patients who needed emergency interventions during the study.

### Assessment

Each patient underwent the following assessments:

1. Demographic Characteristics and Lab Data: Participant demographics (age, sex, education) and relevant laboratory data associated with comorbidities were recorded. Hyperglycemia was defined as a fasting blood glucose level of > 125 mg/dL.
2. Frailty Assessment: Patients were categorized into frail (*n* = 58) and non-frail (*n* = 112) groups based on the Frail Scale and Clinical Frail Scale:
  - Frail Scale: Utilizing a questionnaire developed by The International Association of Nutrition and Aging, individuals were classified as non-frail, pre-frail, or frail based on responses to five questions related to fatigue, resistance, ambulation, illness, and weight loss (FRAIL). Scores of 0, 1–2, and 3–5 indicated non-frail, pre-frail, and frail states, respectively [14].
  - Clinical Frail Scale: Adapted from the Canadian Study of Health and Aging, this scale, comprising nine levels, facilitated clinical assessment of frailty based on a patient’s overall health and life expectancy [15].

Patients were considered frail if any of these two indexes categorized individuals as frail.

3. Functional Assessment: Functional performance was evaluated using the Lawton Instrumental Activities of Daily Living Scale (IADL Lawton):
  - IADL Lawton: This scale gauges eight different instrumental activities of daily living, such as telephone use, shopping, housekeeping, etc. These activities are considered more complicated than normal daily living activities measured in the previous scale. Higher scores in this test indicate greater functional ability [16].
4. Cognitive Assessment: Cognitive function was measured using the Montreal Cognitive Assessment (MOCA) and the Geriatric Depression Scale (GDS):
  - MOCA: A sensitive tool for detecting mild cognitive impairment (MCI), the MOCA evaluates cognitive domains, including attention, executive functions, and memory. Scores range from 0 to 30, with scores of 26 or higher considered normal [17].
  - GDS: The GDS assesses depression in older adults using 15 Yes/No questions and is a very efficient and beneficial method that could increase the detection rate of depression among the elderly population. Scores above 8 suggest depression [18].

### Statistical analysis

Continuous variables are presented as mean and standard deviation (SD) or median and interquartile range (IQR), while categorical or ordinal variables are presented as numbers and percentages. Statistical analyses comparing the two groups were performed using an independent t-test or Wilcoxon rank-sum test for continuous variables and Pearson’s Chi-squared test or Fisher’s exact test for categorical variables, as appropriate. A logistic regression model was performed to assess the association of frailty with the changes in MOCA, GDS, and IADL Lawton scores (Two categories of (1) enhanced and (2) worsened and without change were defined to determine the change status in these scores) adjusted for other clinical and demographic characteristics. Also, in small sample sizes and sparse data settings, Firth-correction logistic regression was used to overcome bias and instability in parameter estimates. The results were presented as odds ratio (OR) with a 95% Confidence Interval (CI). Analyses were conducted using the R Statistical language (version 4.3.0; R Core Team, 2023), using the packages ggstatsplot (version 0.11.1; Patil I, 2021), forcats (version 1.0.0; Wickham H, 2023) and tidyr (version 1.3.0; Wickham H et al., 2023) at 95% confidence interval level (*p* < 0.05) [19–22].

## Results

### Baseline characteristics

This study enrolled a total of 170 patients who underwent elective Coronary Artery Bypass Grafting (CABG) at Tehran Heart Center between 2020 and 2022. The population was predominantly male (75.3%), with a median age of 66 ± 4 years. Only 32% of the patients had a high school diploma or higher education. Most of our patients were overweight, with an average BMI of 26.06 kg/m^2. Additional clinical features are provided in Table 1.

**Table 1:** Baseline characteristic

Characteristic	Overall, *N* = 170^†^	Non-Frail, *N* = 112^†^	Frail, *N* = 58^†^	p-value*
Age	66.0 (62.0, 70.0)	66.0 (62.0, 70.0)	66.0 (62.0, 70.8)	0.938
Female	42 (24.7%)	21 (18.8%)	21 (36.2%)	0.012
Education				0.178
Illiterate	40 (23.5%)	22 (19.6%)	18 (31.0%)	
Lower Diploma	74 (43.5%)	49 (43.8%)	25 (43.1%)	
Diploma & Upper	56 (32.9%)	41 (36.6%)	15 (25.9%)	
BMI	26.06$\pm$3.25	26.05$\pm$3.30	26.09$\pm$3.19	0.671
EF	45.0 (40.0, 55.0)	50.0 (40.0, 55.0)	45.0 (40.0, 55.0)	0.259
CAG				> 0.999
2VD	11 (6.5%)	7 (6.3%)	4 (6.9%)	
3VD	159 (93.5%)	105 (93.8%)	54 (93.1%)	
**Lab data**				
HB	13.34 (1.74)	13.60 (1.66)	12.83 (1.78)	0.004
CR	1.10 (0.90, 1.20)	1.10 (0.90, 1.20)	1.10 (0.90, 1.30)	0.487
FBS	100.5 (88.3, 136.8)	99.50 (87.0, 127.8)	104.5 (91.3, 146.0)	0.179
Blood glucose level				0.241
Normoglycemic	121 (71.2%)	83 (74.1%)	38 (65.5%)	
Hyperglycemic	49 (28.8%)	29 (25.9%)	20 (34.5%)	
TG	114.0 (92.0, 150.0)	110.5 (90.0, 140.8)	120.0 (94.3, 157.8)	0.359
CHOL	157.75$\pm$37.39	162.38$\pm$37.00	148.81$\pm$36.82	0.020
HDL	37.66$\pm$6.96	38.37$\pm$6.59	36.29$\pm$7.50	0.007
LDL	93.81$\pm$24.86	95.54$\pm$24.84	90.48$\pm$24.78	0.183

BMI: Body Mass Index; EF: Ejection fraction; CAG: Coronary artery angiography; 2VD: two-vessel disease;

3VD: three-vessel disease; HB: Hemoglobin; CR: creatinine; FBS: Fasting blood sugar; TG: Triglycerides;

CHOL: Cholesterol; HDL: high-density lipoprotein; LDL: low-density lipoprotein

^†^n (%); Mean$\pm$SD; Median (IQR)

*Pearson’s Chi-squared test; Wilcoxon rank sum test; independent t-test

**Fig. 1:**
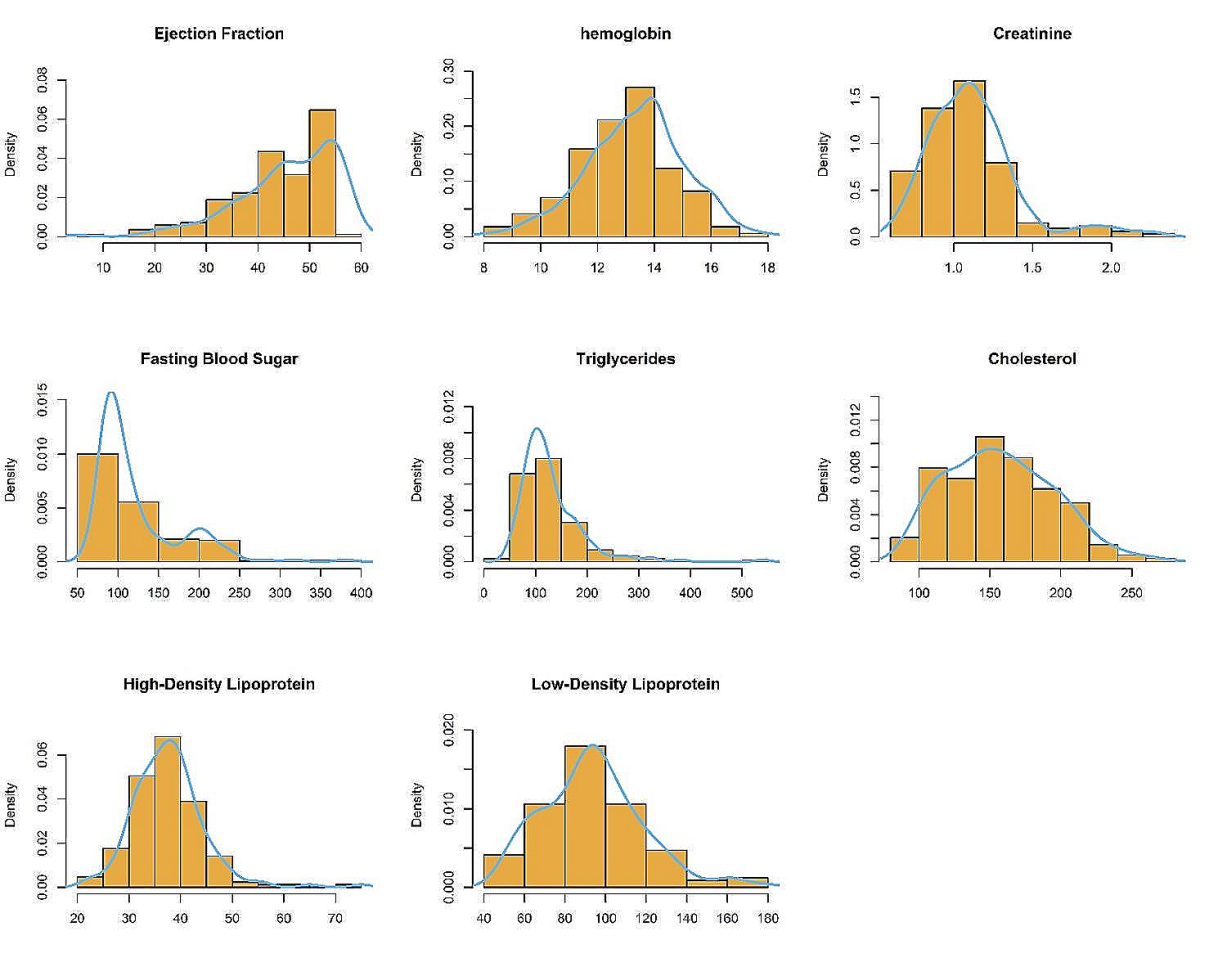
Distribution of patients’ biomarkers

Frail patients had lower ejection fractions (45.00 vs. 50.00, *P* = 0.259), and laboratory results showed decreased levels of Hemoglobin (12.83 vs. 13.60, *P* = 0.004), Cholesterol (148.81 vs. 162.38, *P* = 0.020), and HDL (36.29 vs. 38.37, *P* = 0.007) in frail cases. However, fasting blood sugar (104.5 vs. 99.5, *P* = 0.179) and the prevalence of hyperglycemia (34.5% vs. 25.9%, *P* = 0.241) were not statistically different between the two groups. Figure 1 illustrates the distribution of biomarkers such as lipid profile, hemoglobin, creatinine, fasting blood sugar, and ejection fraction among patients.

**Fig. 2:**
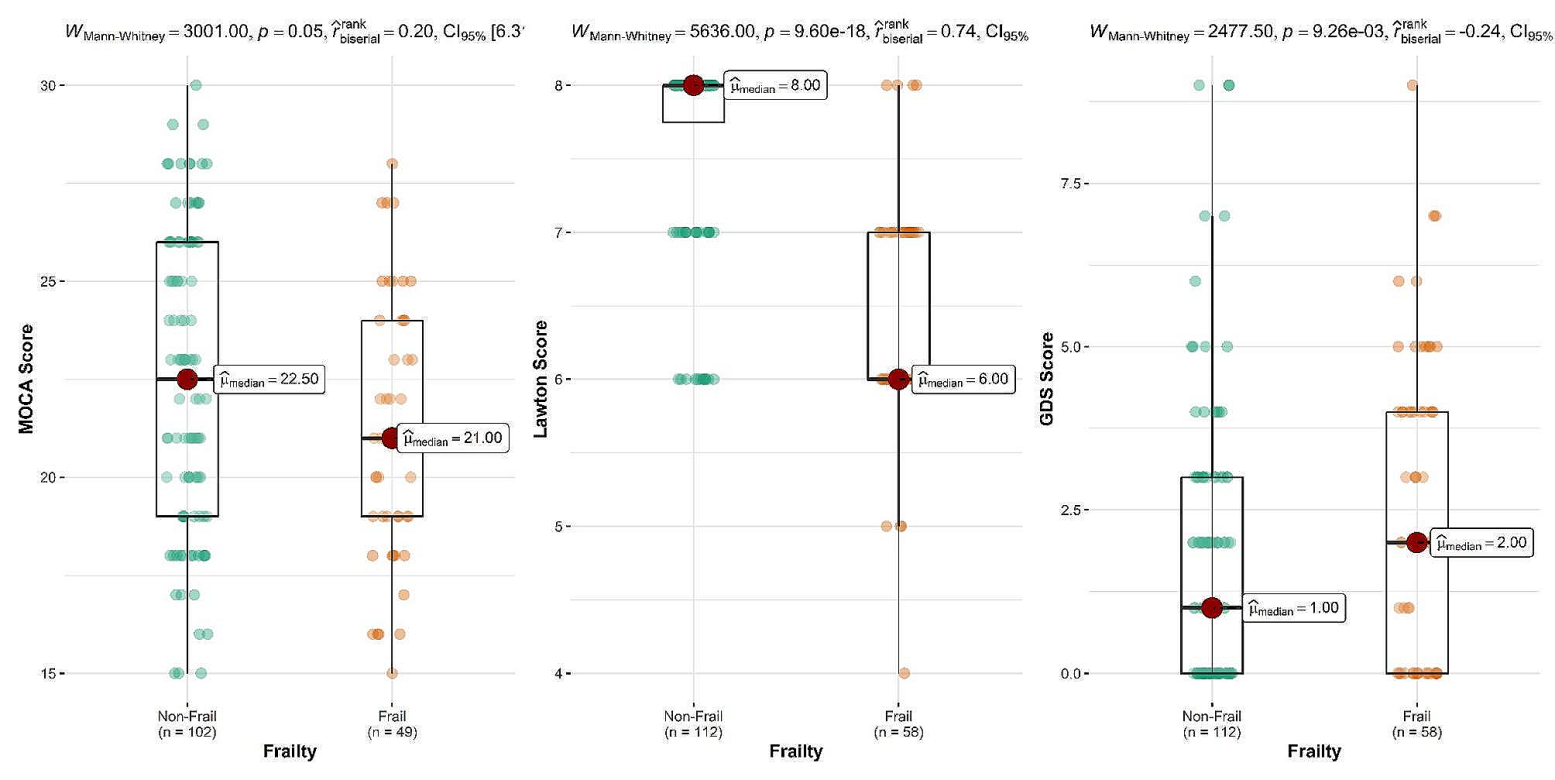
Distribution of preoperative cognitive scores between frail and non-frail groups

Based on the scales we used, 58 patients (34.11%) were classified as frail. Dividing patients’ characteristics by the clinical frailty scale (Supplementary material, Table S1), 60 (35.29%) patients were stage 1–2, 86 (50.58%) were stage 3–4, and 24 (14.11%) were stage 5. According to the patients’ frail scale scores, of the total participants, 110 (64.70%) patients scored 0–1, and 60 (35.29%) patients scored 2–3 (Supplementary material, Table S2). The frail subjects were more frequently male, and most (74.13%) were illiterate or under-diploma. Figure 2 shows the distribution of cognitive scores among patients in each group before surgery.

The frail group’s functional capacity in daily activities seems lower than the non-frail group (6.0 vs. 8.0, *P* < 0.001) (Table 2), as shown by their Lawton scores. Hence, according to the Montreal Cognitive Assessment (MOCA) score evaluation, mild cognitive impairment was seen in both groups. Due to illiteracy, 19 patients could not complete the MOCA questionnaire, so they were not included in this analysis. However, frail patients still displayed lower scores than non-frail subjects (21.0 vs. 22.5, *P* = 0.045). The GDS ratings in frail patients were shown to be higher in comparison to the non-frail group (2.0 vs. 1.0, *P* = 0.009).

**Table 2:** Preoperative and postoperative cognitive and functional scores

Characteristic	Overall, *N* = 170^†*^	Non-Frail, *N* = 112^†*^	Frail, *N* = 58^†*^	p-value**
**Preoperative**				
MOCA	21.0 (19.0, 25.0)	22.5 (19.0, 26.0)	21.0 (19.0, 24.0)	0.045
Lawton	8.0 (6.0, 8.0)	8.0 (7.8, 8.0)	6.0 (6.0, 7.0)	< 0.001
GDS15	2.0 (0.0, 3.0)	1.0 (0.0, 3.0)	2.0 (0.0, 4.0)	0.009
**Postoperative**				
MOCA	23.0 (18.5, 26.0)	23.0 (19.3, 26.0)	22.0 (18.0, 25.0)	0.366
Lawton	7.0 (6.0, 8.0)	8.0 (6.0, 8.0)	6.0 (6.0, 7.0)	< 0.001
GDS15	0.50 (0.0, 2.0)	0.50 (0.0, 2.0)	0.50 (0.0, 2.3)	0.607

^†^Median (IQR)

^*^ 87/170 patients (29/58 of frails and 57/112 of non-frails) were available for follow-up

** Wilcoxon rank sum test

### Primary clinical outcomes

There seems to be a correlation between frailty and complications following the CABG. As indicated in Table 3, patients with frailty were more likely to be readmitted to the hospital (12.1% vs. 1.8%, *P* = 0.008) and also had greater odds of sepsis (19.0% vs. 7.1%, *P* = 0.02) after the surgery and increased rates of EuroSCORE II (1.9 vs. 1.5, *P* < 0.001). The overall death rate was 4.1%, and no association was found between frailty and mortality after the surgery. Frail patients tended to have a longer duration of ICU stay. However, the difference was not statistically significant (6.0 vs. 5.0, *P* = 0.051).

**Table 3:** Postoperative Clinical Outcome

Characteristic	Overall, *N* = 170^†^	Non-Frail, *N* = 112^†^	Frail, *N* = 58^†^	p-value*
Length of Ward Hospitalization	7.0 (5.0, 10.0)	7.0 (5.0, 10.0)	8.00 (6.0, 11.8)	0.100
Length of ICU Hospitalization	6.0 (4.0, 7.0)	5.0 (4.0, 7.0)	6.0 (5.0, 8.0)	0.051
pneumonia	14 (8.2%)	8 (7.1%)	6 (10.3%)	0.559
AF	82 (48.2%)	52 (46.4%)	30 (51.7%)	0.512
Death	7 (4.1%)	4 (3.6%)	3 (5.2%)	0.691
Readmission	9 (5.3%)	2 (1.8%)	7 (12.1%)	0.008
sepsis	19 (11.2%)	8 (7.1%)	11 (19.0%)	0.020
Euroscore	1.7 (1.3, 2.0)	1.5 (1.3, 1.9)	1.9 (1.5, 2.4)	< 0.001

ICU: intensive care unit; AF: Atrial fibrillation

^†^ n (%); Median (IQR)

*Wilcoxon rank sum test; Fisher’s exact test; Pearson’s Chi-squared test

### Cognitive changes after CABG

After being discharged, all patients were contacted by phone to follow up. Out of the total cases, seven patients died, and 86 patients made follow-up postoperative appointments three months after their surgery, so only these patients filled out the questionnaire to assess cognitive and functional impairments. Demographic and clinical characteristics were compared between the loss to follow-up patients and cases with available follow-up data. All clinical, demographic, and frailty characteristics were comparable between the two groups, except for education level, which showed the rate of being highly educated was higher in the follow-up group (*P* = 0.022) (Supplementary material, Table S3). 7 out of 86 patients were illiterate; thus, they were omitted from the MOCA scores change analysis. It is observed that there was no significant difference in the median range of MOCA scores in frail patients following the surgery compared to non-frails. (22.0 vs. 23.0, *p* = 0.366). In addition, there was no considerable difference in the median range of GDS scores between the two groups (0.5 vs. 0.5, *P* = 0.607).

On the other hand, there was a significant difference between frail and non-frail patients in terms of postoperative IADL-Lawton scores, suggesting that frail patients had lower scores (6.0 vs. 8.0, *P* < 0.001).

Based on logistic regression analysis, frailty was not associated with changes in MOCA (OR, 1.34; 95%CI: [0.50, 3.51]; *P* = 0.556), Lawton (OR, 1.33; 95%CI: [0.17, 8.51]; *P* = 0.760), or GDS (OR, 0.32; 95%CI: [0.05, 1.32]; *P* = 0.160) scores (Supplementary material, Table S4).

In addition, an analysis was performed to investigate the association between other factors such as sex, EuroScore, levels of HB and HDL, and complications after the surgery, like sepsis and readmission. There appears to be a correlation between sex (female) and a change in GDS score (OR, 4.29; 95%CI: [1.20, 15.3]; *P* = 0.023). No significant correlation was discovered in this observation between the studied variables and the evaluated cognitive or functional scores (Supplementary material, Table S4).

## Discussion

Our study demonstrated that the clinical outcome of CABG is affected by preoperative frailty. Frail cases showed a significantly higher readmission rate and postoperative complications, such as sepsis. Moreover, the frail group Euroscore was significantly higher, highlighting the importance of frailty application as a risk assessment tool in patients undergoing CABG, which suggests the finding of Shields et al. [23] that combining frailty and Euroscore for risk assessment can improve the prediction of one-year outcome in patients undergoing Percutaneous coronary intervention (PCI) may be generalized to other cardiac intervention, mainly CABG due to result of this study. Furthermore, this group of patients demonstrated a significantly lower baseline MOCA as well as GDS15 and Lawton IADL, representing worse functional, psychological, and independence status. The same worse outcome was observed in these patients concerning postoperative independence level measured by IADL Lawton 3 months after the operation.

Concerning baseline laboratory data, our study demonstrated a significantly lower hemoglobin and cholesterol level in frail patients, which accords with the results of a previous cohort study, including more than 6100 cases undergoing CABG [24]. While our result showed a relatively higher prevalence of hyperglycemia and elevated FBS level, this difference did not reach the significance level, which is in agreement with the results of the Ad et al. study [25] that reported a comparable prevalence of diabetes mellitus in frail and non-frail patients undergoing CABG. However, this contradicts the findings of several previous large-sample studies that demonstrated a significant association between hyperglycemia and frailty [26, 27]. This controversy could be attributed to the small sample size and different frailty criteria utilized in our study and Ad et al. [25], which was not similar to the other mentioned investigations. Furthermore, previous studies illustrated a weaker association between frailty and FBS compared to the 75 gr oral glucose tolerance test result [28]. This could further explain the insignificant association observed in our study, as only FBS has been measured in the included cases. It is noteworthy that baseline hyperglycemia in both diabetic and non-diabetic cases has been shown to be independently associated with adverse outcomes such as restenosis after STEMI treated by stent implantation [29]. In addition, baseline stress hyperglycemia has been shown to have a considerable association (hazard ratio of 2.719) with rehospitalization in patients with Ischemia with nonobstructive coronary arteries [30]. These findings highlight the importance of plasma glucose-related indexes in determining different cardiac diseases, which should be widely investigated in future studies in diabetic and non-diabetic populations.

The higher rate of readmission in frail cases in our study is consistent with the results of Lim et al. [31] study, which reported an odds ratio of 2.58 for readmission in the frail group. These findings may explain the higher cost of postoperative care in frail patients [32] and point to the clinical and financial importance of interventions to reduce the readmission rate in these patients. Regarding this issue, Sarkar et al. [33] proposed a telehealth program for monitoring frail patients’ blood pressure and recovery after cardiac surgery. This program led to an acceptable reduction in hospital readmissions and increased patient satisfaction. Moreover, a longer length of ICU stay in frail participants was observed in our investigation, which was marginally significant(P-val = 0.051). While this finding corroborates the result of the Lim et al. [31]study, it does not support the result of the Jung et al. study [34], which reported an insignificant relation between ICU length of stay and frailty. Further studies are needed to address this controversy and determine if the significance of this association is influenced by factors such as population frailty level and type of cardiac intervention, which are different between these two studies.

The result of our study revealed that postoperative sepsis is significantly more incident in frail patients. This accords with the findings of a retrospective large-population study of almost 254 thousand patients undergoing various major surgeries (including gastrointestinal-related operations, joint replacement surgeries, and traumatic hip surgical management), which demonstrated that hospital frailty risk score (HFRS) can be utilized as a valid and accurate risk classification for postoperative sepsis in cases undergoing major surgeries [35]. Our findings suggest that frailty can further serve as a risk assessment tool in patients undergoing CABG and facilitate the early detection of sepsis in high-risk cases. In addition, we suggest further studies to focus on the applicability of frailty as a risk score for sepsis in other cardiac procedures. The importance of this result is further highlighted according to the fact that early diagnosis of sepsis can lead to improved clinical outcomes [36] using an interdisciplinary management approach, which should mainly take advantage of anesthesiology, nutrition, geriatric, and internal medicine [37].

Our investigation demonstrated that the incidence of pneumonia was almost 1.5-fold higher in frail patients. However, the difference between frails and non-frails did not reach statistical significance. A previous study by Henry et al. [38] measured the difference in pneumonia incidence in frails and non-frails, considering two different frailty assessing tools reported (CHS and SOF were administered). They illustrate that the significance of this difference is dependent on the frailty scoring system used to classify patients, as pneumonia incidence was comparable between frails and non-frails using CHS as the frailty scoring instrument (p-value of 0.35); at the same time, it was nine times higher in frails using SOF as the frailty assessment tool (p-value of 0.0014). These findings suggest that patients with frailty should be managed cautiously, considering postoperative pneumonia. However, it should be noted that the relation between sepsis and frailty is more considerable compared to pneumonia, and taking action to manage sepsis is of high priority.

This study showed that no significant association between frailty and 3-month mortality exists. Kochar et al. [39] also reported that short-term (3-month and 1-year) mortality is not correlated with frailty, while long-term (5-year) mortality showed a significant correlation with frailty. This could be explained by the fact that mortality was not the primary outcome of our study (similar to Kochar et al. [39]), and the small number of deaths in these periods might have reduced the statistical power of the analysis. However, this is in contrast to the findings of some previous studies [24, 40], which reported that 1-month mortality is significantly associated with frailty. This may suggest that frailty’s effect on mortality depends on the follow-up period and type of cases included. Further study is needed to evaluate this explanation.

Regarding cognitive function, depression, and independence level, our results revealed that frail patients demonstrate a significantly worse baseline score based on the MOCA, GDS-15, and IADL Lawton (p-values of 0.045, 0.009, and < 0.001, respectively). This is consistent with the results of previous studies [41]. This shows the importance of integrating cognitive and psychological criteria into physical frailty indexes. It has been shown that adding mild cognitive impairment (MCI) to physical frailty considerably affects risk stratification for postoperative delirium (including patients of any cardiac intervention), as the odds ratio increased from 2.5 (in cases with only physical frailty) to 8.6 (in subjects with both frailty and MCI) compared to patients without MCI or physical frailty [41]. Furthermore, the predictive role of baseline depression and cognitive status for postoperative independence has been highlighted in coronary angiography. However, it should be mentioned that different scoring systems for assessing these two baseline characteristics have shown various accuracies for predicting postoperative disability (with an AUC range from 0.53 to 0.62), while combining these factors increased the AUC to 0.76 [42].

Concerning improvement/decline of cognitive function, depression, and independence level, This study revealed no significant differences between frail and non-frail patients at three-month follow-up. Although these results differ from findings of previous studies after 1-month after cardiac surgery [11, 12] (a significantly greater decrease in frail patients, p-value of 0.02,0.49, and 0.01, respectively), it supports their 1-year results that illustrate no significant difference in cognitive decline between frails and non-frails (p-value of 0.36) [11]. It is reasonable to consider the clinical assumption that changes in the cognitive and psychologic function levels are not the same in different follow-up periods. While 1-month results show a greater decrease in frail patients, the following months’ investigations proposed that this difference will be attenuated. This accords with the findings of our study as well as Nomura et al. [11]. and Freiheit et al. [43]. The clinical implication of this trend is to pay attention to the first postoperative month of cognitive care in frail patients and specify the main period in which the efficacy of interventions should be evaluated. Moreover, it proposes how frail patients will regain their cognitive function and can contribute to the monitoring treatment response guidelines.

This investigation unveiled a significantly lower postoperative IADL Lawton score in frail patients (P-value < 0.001). This low functional ability and insignificant difference of cognitive score between frail and non-frail that has been observed in our study are to some extent consistent with the findings of Beska et al. that after invasive management of acute coronary syndrome, frail patients’ physical function was significantly lower while their mental status was the same, measured by the SF-36 scale [44]. Furthermore, frail patients who had undergone vascular surgery were considerably more likely (62% vs. 22%) to require post-operative mobility assistance compared to non-frail [45].

According to our results, the prevalence of the female gender was almost two times higher in a frail group compared to non-frails (P-value of 0.012). Moreover, it was demonstrated that the female gender was considerably associated with GDS score change after CABG (OR = 4.29). These findings support the conclusion of a Mone et al. study [46] that older women are at greater risk of developing frailty. In addition, they reported that the female gender is associated with worse functional and mental status and poor clinical outcomes in patients undergoing PCI due to myocardial infarction. Considering this issue, they suggested performing a 5-meter gait exam and a mini-mental status exam prior to hospital discharge in these cases. The results of this could be beneficial in the precise assessment of patients’ status as well as in designing treatment and rehabilitation plans.

Indeed, our findings underscore the significant association between baseline frailty and postoperative independence level in patients undergoing CABG, an invasive surgery. This suggests that rehabilitation programs should prioritize actions to improve the independence level of frail patients. This insight could be instrumental in enhancing patient care and outcomes following CABG. Concerning resource allocation for patients undergoing CABG, we recommend that frail patients be the focus of postoperative care, specifically regarding assistance demanded activity. Moreover, risk assessment based on frailty level combined with using specific guidelines [47] that have focused on optimizing perioperative management of frails to improve surgery outcomes is of paramount importance, as rehabilitation in elderly patients classified as frail using physical, nutritional, and cognitive-related interventions have been shown to reverse frailty considerably [48].

### Strengths and limitations

The major strength of this study is the precision of gathering data regarding exposures, confounding variables, and outcomes due to its prospective design. Baseline assessment of psychological, cognitive, and independence levels by validated relevant scales prevents the misinterpretation of postoperative results caused by baseline differences. Moreover, all of the included patients underwent CABG in contrast to some previous similar studies that included patients undergoing different cardiac surgeries and considered them as identical [11, 41]. This increases the accuracy of our result to be applied to this group of patients. In addition, the follow-up period of this study is more extended than a similar study measuring perioperative cognitive and independence changes in CABG surgery [11, 12], which can provide a novel insight into the trends of these characteristics in the first month after operation.

However, this study has several limitations. First, a significant proportion of patients missed the three-month follow-up, and cognitive assessment was not available for this group. This could introduce bias and result in a Type II error. However, as illustrated in Table S3, except for the education level, there was no significant difference between the group lost to follow-up and the patients available for follow-up, indicating the validity of our results. Second, several scales have been developed to assess frailty, showing different accuracy in predicting outcomes. In this study, two of them have been utilized, and it should be considered that the results may differ when using other scales. Moreover, it should be mentioned that Fried criteria have been used as one of the main scoring systems to assess frailty [49, 50]. In addition, measured physical and cognitive frailty based on this index have been highlighted as efficient prognostic factors in ACS patients [51]. However, according to the ethical committee decision, measuring hands’ grip force and gait speed in patients with CABG indication was not clinically reasonable because of the severe activity level needed for these processes. Concerning this issue, in this study, we utilized the Frail Scale and Clinical Frail Scale to assess patients’ frailty status. Further studies are necessary to evaluate the consistency of frailty measurement in CABG patients using different scales. Third, this prospective study was conducted in a single center, and the results may not be generalizable to other cardiology intervention centers, necessitating validating these findings in multi-central prospective studies. Fourth, this investigation was conducted using a relatively small sample size with short-term follow-up. This indicates that further large sample size studies are necessary to assess the frailty association with functional and status outcomes with mid-term and long-term follow-up. Fifth, patients’ status regarding some comorbidities such as diabetes mellitus, hypertension, and pulmonary diseases were not precisely determined in past medical history records of the included cases of this study, which should be taken into consideration when interpreting the results of this study. We suggest future investigation to assess the association of these comorbidities with clinical and cognitive outcomes to assess whether integrating these factors with currently available frailty criteria can enhance the predictive values of these scoring systems.

## Conclusion

In conclusion, our study demonstrated that frail patients undergoing CABG have a significantly higher incidence rate of clinical complications, including readmission and sepsis. Moreover, the postoperative Euroscore was significantly higher in frail cases. A considerably more extended ICU stay was also observed in patients with preoperative frailty. However, it is noteworthy that death rates were comparable between the two groups.

According to the GDS15, MOCA, and IADL Lawton, baseline scores in frail patients were significantly worse than in non-frail patients. However, considering the changes in these scores three months after CABG, none were considerably different between the two groups.

While postoperative GDS15 and MOCA were statistically similar between the two groups, IADL Lawton after the surgery was significantly worse in frail patients. This highlights that postoperative care focusing on frail patients’ independence level is paramount and should be considered in health policymaking.

### Electronic supplementary material

Below is the link to the electronic supplementary material.

Supplementary Material 1

## Data Availability

Availability of data and materials: The datasets utilized and/or analyzed in the current investigation are accessible upon reasonable request from the corresponding author.

## Abbreviations

CABG: Coronary Artery Bypass Grafting
GDS: and the Geriatric Depression Scale
MOCA: Montreal Cognitive Assessment
IALD Lawoton: Lawton Instrumental Activities of Daily Living Scale
OR: Odds ratio
CI: Confidence Interval
AF: Atrial Fibrillation
