# Supplementary material for: Frailty efficacy as a predictor of clinical and cognitive complications in patients undergoing coronary artery bypass grafting: a prospective cohort study
Source: BMC Cardiovasc Disord. 2024 Feb 16;24:110. doi: 10.1186/s12872-024-03781-7 (PMC10874082; doi:10.1186/s12872-024-03781-7)
Supplement: Supplementary file 1 — Supplementary Material 1 [file 12872_2024_3781_MOESM1_ESM.docx]

**Table S1. Preoperative Clinical Frailty Scale incidence**

| **Characteristic** | **Overall**,  N = 170^†^ | **Non-Frail**,  N = 112^†^ | **Frail**,  N = 58^†^ |
| --- | --- | --- | --- |
| Clinical stage |  | | |
| Stage 1 | 5 (2.9%) | 5 (4.5%) | 0 (0%) |
| Stage 2 | 55 (32%) | 55 (49%) | 0 (0%) |
| Stage 3 | 52 (31%) | 52 (46%) | 0 (0%) |
| Stage 4 | 34 (20%) | 0 (0%) | 34 (59%) |
| Stage 5 | 24 (14%) | 0 (0%) | 24 (41%) |
| Stage 6 | 0 (0%) | 0 (0%) | 0 (0%) |
| ^†^n (%) | | | |

**Table S2. Preoperative frail scale incidence**

| **Characteristic** | **Overall**,  N = 170^†^ | **Non-Frail**,  N = 112^†^ | **Frail**,  N = 58^†^ |
| --- | --- | --- | --- |
| Frail score |  | | |
| 0 | 78 (46%) | 78 (70%) | 0 (0%) |
| 1 | 32 (19%) | 31 (28%) | 1 (1.7%) |
| 2 | 15 (8.8%) | 2 (1.8%) | 13 (22%) |
| 3 | 45 (26%) | 1 (0.9%) | 44 (76%) |
| 4 | 0 (0%) | 0 (0%) | 0 (0%) |
| ^†^n (%) | | | |

**Table S3. Baseline characteristic comparison between missing cases and followed-up patients**

| **Characteristic** | **Followed-up,**  **N = 86**^†^ | **Loss to follow-up,**  **N = 84**^†^ | **p-value**^★^ |
| --- | --- | --- | --- |
| Age | 66.34 (5.54) | 67.02 (5.38) | 0.319 |
| Frail |  |  | 0.912 |
| Non-Frail | 57 (66.3%) | 55 (65.5%) |  |
| Frail | 29 (33.7%) | 29 (34.5%) |  |
| Female | 19 (22.1%) | 23 (27.4%) | 0.424 |
| Education |  |  | 0.022 |
| Illiterate | 13 (15.1%) | 27 (32.1%) |  |
| Lower Diploma | 39 (45.3%) | 35 (41.7%) |  |
| Diploma & Upper | 34 (39.5%) | 22 (26.2%) |  |
| BMI | 26.26$\pm$3.29 | 25.87$\pm$3.21 | 0.346 |
| EF | 50.0 (40.0, 55.0) | 45.0 (40.0, 55.0) | 0.246 |
| **Lab data** |  |  |  |
| HB | 13.0 (12.9, 14.0) | 13.5 (12.0, 14.0) | 0.717 |
| CR | 1.10 (0.90, 1.20) | 1.10 (1.00, 1.30) | 0.136 |
| FBS | 103.5 (92.0, 136.8) | 98.0 (87.0, 137.8) | 0.313 |
| TG | 120.0 (95.0, 158.0) | 112.0 (89.0, 135.5) | 0.265 |
| CHOL | 157.17 ± 37.00 | 158.33 ± 38.00 | 0.847 |
| HDL | 37.66 ± 6.90 | 37.65 ± 7.07 | 0.757 |
| LDL | 94.31 ± 23.77 | 93.30 ± 26.06 | 0.557 |
| ^†^n (%); Mean$\pm$SD; Median (IQR)  ^★^Pearson's Chi-squared test; Wilcoxon rank sum test; independent t-test | | | |
| (BMI: Body Mass Index; EF: Ejection fraction; HB: Hemoglobin; CR: creatinine; FBS: Fasting blood sugar; TG: Triglycerides; CHOL: Cholesterol; HDL: high-density lipoprotein; LDL: low-density lipoprotein) | | | |

**Table S4. Cognitive and functional scores change after the operation**

|  |  | **MOCA** | | | |  | **Lawton** | | | |  | **GDS** | | | |
| --- | --- | --- | --- | --- | --- | --- | --- | --- | --- | --- | --- | --- | --- | --- | --- |
| **Characteristic** |  | **N** | **OR**^†^ | **95% CI**^†^ | **p-value** |  | **N** | **OR**^†^ | **95% CI**^†^ | **p-value** |  | **N** | **OR**^†^ | **95% CI**^†^ | **p-value** |
| Frai vs non-frail |  | 79 | 1.34 | 0.50, 3.51 | 0.556 |  | 86 | 1.33 | 0.17, 8.51 | 0.760 |  | 85 | 0.32 | 0.05, 1.32 | 0.160 |
| Female vs Male |  | 79 | 0.52 | 0.15, 1.56 | 0.262 |  | 86 | 0.88 | 0.04, 6.39 | 0.908 |  | 85 | 4.29 | 1.20, 15.3 | 0.023 |
| HB |  | 79 | 1.25 | 0.94, 1.70 | 0.144 |  | 86 | 0.94 | 0.56, 1.65 | 0.821 |  | 85 | 0.77 | 0.54, 1.09 | 0.136 |
| HDL |  | 79 | 0.99 | 0.92, 1.06 | 0.741 |  | 86 | 0.98 | 0.84, 1.11 | 0.721 |  | 85 | 1.03 | 0.94, 1.12 | 0.526 |
| Readmission |  | 79 | 0.50 | 0.02, 4.11 | 0.56 |  | 86 | 1.26 | 0.01, 13.9 | 0.882^*^ |  | 85 | 0.45 | 0.01, 4.39 | 0.563^*^ |
| sepsis |  | 79 | 0.63 | 0.13, 2.47 | 0.525 |  | 86 | 1.44 | 0.07, 10.8 | 0.755 |  | 85 | 0.16 | 0.01, 1.38 | 0.110^*^ |
| Euroscore |  | 79 | 1.75 | 0.82, 3.97 | 0.155 |  | 86 | 1.43 | 0.38, 3.74 | 0.507 |  | 85 | 1.04 | 0.40, 2.25 | 0.924 |
| ^†^OR: Odds Ratio, CI: Confidence Interval  ^*^Firth correction  (HB: Hemoglobin; HDL: high-density lipoprotein) | | | | | | | | | | | | | | | |
